# Human ADMC-Derived Adipocyte Thermogenic Capacity Is Regulated by IL-4 Receptor

**DOI:** 10.1155/2017/2767916

**Published:** 2017-10-12

**Authors:** Fernando Lizcano, Diana Vargas, Ángela Gómez, Astrid Torrado

**Affiliations:** Center of Biomedical Investigation from University of La Sabana (CIBUS), Chía, Cundinamarca, Colombia

## Abstract

Type two innate immune system is anti-inflammatory and may play an important role as the means whereby “browning” is induced in subcutaneous adipocytes. It was shown that IL-4 may influence the fate of adipose cell precursors by promoting differentiation towards more thermogenic adipocytes in mice. Here, we investigated the influence of IL-4 and IL-4 receptor, a type two immune cytokine pathway, on the metabolic activity and thermogenic potential of human adipocytes differentiated from adipose-derived mesenchymal stem cells (ADMSCs) obtained from subcutaneous samples of healthy women undergoing abdominoplasty. Western blot analysis, qPCR, and biochemical analyses were performed 10 days after ADMSC differentiation into mature adipocytes was induced. IL-4 receptor was expressed in both precursor and differentiated adipocytes, and IL-4 treatment increased phosphorylation Y641 of signal transducer and activator of transcription 6 (STAT6) in both cell types. IL-4 treatment also increased expression of thermogenic proteins PGC-1*α*, UCP-1, and CITED1. In addition, IL-4 increased the secretion of adiponectin, leptin, and FGF21 and promoted lipolysis in differentiated adipocytes. In conclusion, IL-4 may directly modulate differentiation of human adipocytes towards a beige phenotype acting through IL-4 receptors on both adipose precursors and differentiated human adipocytes, metabolic effect that must be considered in some antiallergic drugs.

## 1. Introduction

Obesity is associated with a state of mild inflammation, which can contribute to insulin resistance and type 2 diabetes mellitus [1]. This mild inflammatory condition is characterized by changes in the macrophage population in adipose tissue [2, 3]. An increase in proinflammatory type-1 macrophages (M1), which promotes insulin resistance, is evident in the adipose tissue of obese people, who also demonstrate reduced numbers of type-2 macrophages (M2), which are insulin sensitizing [4, 5].

Several studies have emphasized the role of interleukins (ILs) in the physiology of adipose cells: ILs increase lipolysis and modify body fat distribution and production of extracellular matrix by adipose tissue [6–8]. However, it was suggested that the interaction between adipose cells and the immune system is more complex than previously thought [9]. Some studies have shown that the activation of group 2 innate lymphoid cells (ILC2s) may modify the function of adipose tissue and systemic glucose homeostasis. It was observed that after IL-33 stimulation, eosinophils and ILC2s produce IL-4 and IL-13 [10–13], which are ILs that may promote the differentiation of adipose precursors into “brown like” adipocytes [14]. Additionally, IL-33 can influence the production of met-enkephalin by ILC2s, which can induce the process of “browning” in differentiated adipose cells [10]. Additionally, M2 macrophages that are activated by IL-4 can be mobilized in the subcutaneous adipose tissue, secreting catecholamines that activate brown adipocytes and induce the differentiation of beige adipocytes [14, 15]. We previously observed an elevation of thermogenic gene expression induced by cold that was increased by IL-4 in human adipocytes [16].

Despite the observed effect of IL-4 on the differentiation of beige adipocytes from precursor cells, the effects of IL-4 on differentiated adipocytes and its influence on the process of browning have yet to be investigated. Previous studies showed reduced IL-4 receptor (IL-4R) expression in differentiated adipose cells. However, these studies were primarily performed in 3 T3-L1 cells and mouse adipose tissue [11, 17]. Therefore, the functional roles of IL-4 and its receptor remain to be determined in human differentiated adipocytes. On the other hand, it recently was observed that the adaptive thermogenesis is not commonly associated to a chronic treatment with IL-4 in mice. Fischer et al. reported opposite results with respect to the previous assessment of relevant amounts of catecholamine synthesis by IL-4 alternatively activated macrophages, which involved a role of IL-4 in adaptive thermogenesis and brown/beige adipocyte metabolism [18]. The regular signaling pathway of IL-4 is initiated by binding of ligand to type I receptors (IL-4R*α*/*γ*-chain) and type II receptors (IL-4R*α*/IL-13R*α*1) [19]. The binding of IL-4 to IL-4R induces receptor dimerization, which activates an intracellular signaling pathway [20] that involves the activation of the transcription factor signal transducer and activator of transcription 6 (STAT6) and insulin receptor substrate 2 [21, 22].

To add to the limited knowledge regarding the influence of IL-4 on the browning process in humans, we studied the phenotypic characteristics of differentiated adipocytes from healthy women undergoing lipectomy. We observed that IL-4 treatment induced phenotypic characteristics typical of beige adipocytes in differentiated white adipocytes. Additionally, we found that both precursor cells and differentiated adipocytes express IL-4R, and IL-4 induces an increase in phosphorylation of STAT6. This implies that IL-4 may have a direct effect on differentiated adipocytes through IL-4R, resulting in “browning.”

## 2. Materials and Methods

Subcutaneous fat samples were obtained from eight healthy women aged 20–40 years, who were undergoing abdominoplasty. They had body mass indexes (BMIs) of 23–25 kg/m^2^ and had received no drug treatment during the 3 months prior to sampling and did not show any signs of disease. In addition, their lipid profiles and glucose levels were within the normal range. They received detailed information regarding the purpose of the study and gave informed consent. The project was approved by the ethics committee of the University of La Sabana.

### 2.1. Cell Culture and Differentiation

Precursor cells were isolated from 30 g abdominal subcutaneous adipose tissue obtained during surgery. Adipose samples were washed with phosphate-buffered saline (PBS), and all visible fibrous material and blood vessels were removed. Subsequently, samples were digested with 250 U/mL type I collagenase, 20 mg/mL bovine serum albumin (BSA), and 60 *μ*g/mL gentamicin in PBS for 90 min at 37°C. After digestion, the samples were centrifuged at 200 ×g for 10 min, and the pellet was resuspended in an erythrocyte lysis solution containing 154 mM ammonium chloride, 5.7 mM monobasic potassium phosphate, and 0.1 mM EDTA pH 7.3 for 10 min. This mixture was filtered through a nylon mesh (pore size: 150 *μ*m), followed by centrifugation at 200 ×g for 10 min. The cell pellet was then resuspended in proliferation media containing Dulbecco's Modified Eagle Medium (DMEM)/F12, 10% *v*/*v* fetal bovine serum, and 50 *μ*g/mL gentamicin and seeded at a density of 10,000 cells/cm^2^.

After 24 h, the cells were washed and induced to proliferate by addition of PM4 media (DMEM/F12 containing 2.5% fetal bovine serum, 1 ng/mL basic fibroblast growth factor, 10 ng/mL epidermal growth factor, and 8.7 *μ*M insulin). When cells had reached 100% confluency, human precursor cells were differentiated into adipocytes by addition of DMEM/F12 medium containing 66 nmol L^−1^ insulin, 1 nmol L^−1^ triiodo-L-thyronine, 10 *μ*g/mL transferrin, 0.5 mmol L^−1^ isobutyl-methylxanthine, 100 nmol L^−1^ dexamethasone, and 1 *μ*mol L^−1^ rosiglitazone (a peroxisome proliferator-activated receptor *γ* (PPAR*γ*) agonist) for 72 h. Subsequently, the medium was replaced with preadipocyte basal medium containing the same concentrations of insulin, triiodo-L-thyronine, and transferrin for 10 days, with changes every 3 days. The effect of IL-4 on mature adipocytes was evaluated by treatment with 10 ng/mL of IL-4 (PeproTech, Rocky Hill, NJ, USA) in 0.1% *w*/*v* BSA for 6 h. As a negative control, mature adipocytes were treated with 100 ng/mL IL6 (Cell Signaling Technology, Danvers, MA, USA) in 0.1% *w*/*v* BSA for 6 h.

### 2.2. Adipocyte Cold Induction

In previous studies, performed by our group, primary human adipose cells were evaluated and observed that a reduction on temperature from 37°C to 30°C during a period may resemble a cold induction with an increase of thermogenic proteins, UCP-1 and PGC-1*α*.

Adipocyte precursor cells were induced to differentiate by culturing in 6-well plates for 10 days at 37°C in basal differentiation medium. Cells were washed with PBS, basal medium was added, and then cells were incubated at 30°C in 5% CO_2_ and in the presence or absence of 10 ng/mL IL-4 for 6 h. Cells were then lysed for analysis for protein expression. Control cells were cultured under the same conditions at 37°C [16].

### 2.3. qPCR

Total RNA was isolated from both human adipocyte precursor cells and differentiated adipocytes. RNA extraction was performed using a High Pure RNA Isolation Kit (Roche Diagnostics, Mannheim, Germany) in accordance with the manufacturer's instructions. Then, 500 ng RNA was used to generate cDNA using a Transcriptor First Strand cDNA Synthesis Kit (Roche Diagnostics, Mannheim, Germany). The following primers were used for gene expression detection, following the recommended protocol for use with the FastStart Essential DNA Green Master Mix (Roche Diagnostics, Mannheim, Germany): IL-4R Fw: 5′ GTGCTATGTCAGCATCACCAAGA 3′, Rev: 5′ CCCCTGAGCATCCTGGATTAT 3′ and uncoupling protein 1 (UCP-1) Fw: 5′ GTGTGCCCAACTGTGCAATG 3′, Rev: 5′ CCAGGATCCAAGTCGCAAGA 3′. Quantitative analyses of mRNA expression levels were normalized to expression of glyceraldehyde-3-phosphate dehydrogenase (GAPDH; primer sequences: Fw 5′ ACCCACTCCTCCACCTTTGAC 3′ and Rev 5′ TGTTGCTGTAGCCAAATTCGTT 3′ using the ΔΔCt method.

### 2.4. Western Blot Analysis

Adipocytes differentiated from subcutaneous adipose tissue were lysed in radioimmunoprecipitation assay (RIPA) buffer (Abcam, Cambridge, MA, USA; ab156034) containing 1 *μ*g protease inhibitor cocktail (Roche Diagnostics, Mannheim, Germany). Total protein content of each lysate was then quantified using the Bradford method, and the lysates were diluted to a final working concentration of 50 *μ*g/mL protein. Lysates were denatured at 95°C and subjected to polyacrylamide gel electrophoresis. Products were then electrotransferred to polyvinylidene difluoride membranes pretreated with 100% methanol for 2 min. Blocking was performed in PBS-T (1x PBS containing 0.1% Tween 20) containing 5% *w*/*v* skimmed milk powder.

Membranes were then incubated with antibodies targeting proteins involved in thermogenesis: rabbit anti-PGC-1*α* (1 : 1000, Abcam ab54481), rabbit anti-UCP-1 (1 : 1000, Abcam ab155117), rabbit anti-IL-4R (1 : 1000, Abcam ab131058), rabbit anti-STAT6 phosphorylated (phospho Y641) (1 : 1000, Abcam ab54461), and mouse anti-cAMP response element-binding protein/p300-interacting transactivator 1 (CITED1; Abcam ab87978). Secondary antibodies against rabbit IgG, conjugated to horseradish peroxidase (IgG-HRP) for PGC-1*α*, IL-4R, and pSTAT6 (1 : 5000) and UCP1 (1 : 3000), were used. Mouse IgG-HRP was used as the secondary antibody following anti-CITED1 application (1 : 2000).

Expression levels of adipokines were measured using rabbit anti-fatty acid-binding protein 4 (FABP4; 1 : 3000, Abcam 92,501), anti-adiponectin (1 : 3000, Abcam ab92501), fibroblast growth factor 21 (FGF21; 1 : 1000, Abcam ab171941), and leptin (Abcam ab16227), with rabbit IgG-HRP (1 : 5000) being used as a secondary antibody. Detection was performed by chemiluminescence using the Luminata Crescendo Kit (Millipore, Billerica, MA, USA). Images were captured and analyzed using myECL Imager, a blot and gel documentation instrument (Catalog number 62236, Thermo Scientific, Waltham, MA, USA). Quantitative densitometric analysis was performed using My Image Analysis software for three independent experiments.

### 2.5. Lipolysis Assay

Precursors and differentiated adipocytes were washed and incubated in assay buffer in the presence of BSA as a carrier or were treated with 10 ng/mL IL-4. As a positive control, cells were treated with 1 *μ*M isoproterenol. Glycerol content of the supernatant was measured using a Cultured Human Adipocyte Lipolysis Assay Kit (Zen-Bio, Research Triangle Park, NC, USA) following the manufacturer's instructions, with spectrophotometric detection at 540 nm.

### 2.6. Statistical Analysis

Data were evaluated using one-way ANOVA, data represent means ± SD of three independent experiments from the samples of 8 patients which proteins were analyzed by Western blot. The differences between means were performed through Student's *t*-test. Differences were considered statistically significant when *p* < 0.05.

## 3. Results

### 3.1. IL-4 Activates the IL-4R Signaling Pathway in Precursors and Differentiated Adipocytes

This study builds on previous experiments in which mouse adipocyte precursors treated with IL-4 demonstrated a beige phenotype, following activation of the IL-4R/STAT-6 pathway. By contrast, differentiated adipocytes were thought to undergo “browning” because of the activation of a different pathway, because they do not express IL-4R [11, 14]. Adipose-derived mesenchymal stem cells (ADMSCs) from human subcutaneous fat were induced to differentiate for 10 days. The expression of IL-4R was measured in adipocyte precursors (stromal vascular fraction (SVF)) and adipocytes were induced to differentiate using a differentiation cocktail (mature adipocytes), before and after treatment with IL-4.

**Figure 1:**
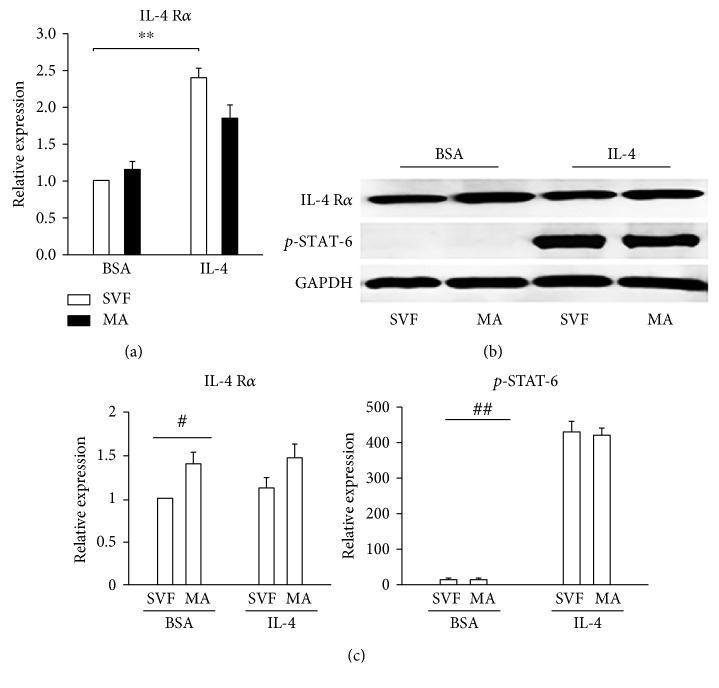
Expression of IL-4R*α* and pSTAT-6 in precursor and mature adipocytes. Precursor cells (SVF) were obtained from human subcutaneous adipose tissue, induced to proliferate, and then differentiated into mature adipocytes (MA), when they were treated with BSA alone (vehicle) or IL-4. (a) IL-4R mRNA expression was quantified by qPCR using GAPDH as a reference gene. Data represent mean ± SD. ^∗∗^*p* < 0.01. (b) IL-4R and phosphorylated STAT-6 (pSTAT-6) protein were measured using Western blot. (c) Relative band intensity was determined by densitometry. Statistical analyses were performed using ANOVA. Data represent mean ± SD. ^#^*p* < 0.05 between precursors (SVF) and differentiated adipocytes (MA) after 6 h of BSA or IL-4 treatment. ^##^*p* < 0.001 between precursors (SVF) and differentiated adipocytes (MA) before and after 6 h of IL-4 treatment. Data were normalized to GAPDH as reference gene/protein.

As shown in Figure 1(a), both precursor and mature adipocytes expressed IL-4R mRNA and IL-4 treatment significantly increased the expression of IL-4R in both types of cells. Previous studies showed that IL-4 can induce browning in precursor adipocytes through IL-4R [11]. However, IL-4R was shown not to be expressed by adipocytes derived from murine precursor cells. We aimed to evaluate the role of IL-4R in differentiated adipocytes from human subcutaneous adipose tissue; therefore, protein levels of IL-4R and pSTAT-6 were determined. IL-4R protein was expressed in both precursors and differentiated adipocytes, and pSTAT-6 was increased by the administration of IL-4 to both cell types (Figures 1(b) and 1(c)).

### 3.2. pSTAT-6 Is Induced by IL-4 in Mature Adipocytes

**Figure 2:**
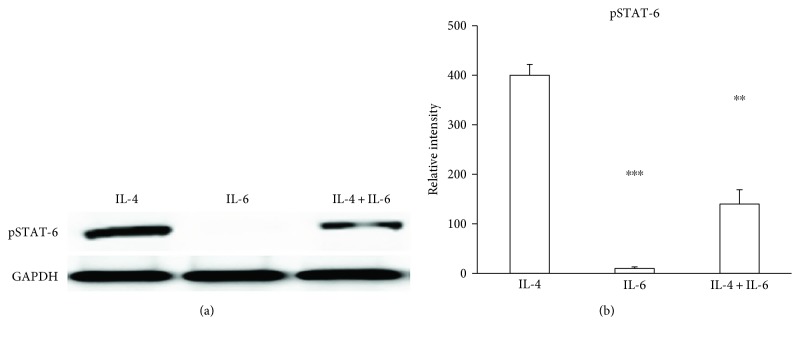
pSTAT-6 is induced by IL-4 treatment of mature adipocytes. Precursor cells were obtained, proliferated, and induced to differentiate into mature adipocytes, followed by treatment with BSA alone (vehicle), IL-4, 10 ng/mL; IL-6, 100 ng/mL, or IL-4, 10 ng/mL and IL-6, 100 ng/mL. (a) Phosphorylated STAT-6 (pSTAT-6) protein was measured using Western blot. (b) Phosphorylated STAT-6 (pSTAT-6) protein expression was measured by Western blotting. Relative band intensity was determined by densitometry. Statistical analyses were performed using ANOVA. Data represent mean ± SD. ^∗∗^*p* < 0.01 between mature adipocytes treated with IL-4 and IL-4 + IL-6. ^∗∗∗^*p* < 0.001 between mature adipocytes (MA) treated with IL-4 or IL-6.

To establish whether the IL-4 signaling pathway is activated following IL-4 treatment of mature adipocytes, we assayed pSTAT-6. Mature adipocytes were treated with IL-4, IL-6, or IL-4 and IL-6. As expected, pSTAT-6 was detected in adipocytes treated with IL-4, while IL-6 treatment did not induce pSTAT-6 (Figures 2(a) and 2(b)).

### 3.3. Effect of IL-4 on Thermogenic Protein Expression

**Figure 3:**
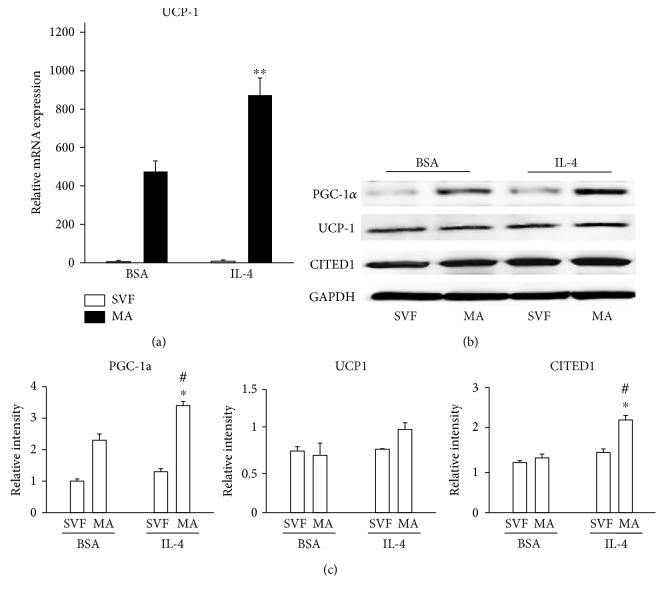
Effect of IL-4 on the expression of thermogenic genes. Precursor cells were obtained, proliferated, and induced to differentiate into mature adipocytes and then treated with BSA alone (vehicle) or IL-4. (a) UCP-1 mRNA expression was measured by qPCR. (b) Protein expression of PGC-1*α*, UCP-1, and CITED1 was measured by Western blotting. (c) Relative band intensities for each were determined by densitometry. Statistical analyses were performed using ANOVA. Data represent mean ± SD and were normalized to GAPDH as reference gene/protein. ^∗^*p* < 0.05 between precursors (SVF) and mature adipocytes (MA) after 6 h of IL-4 treatment, ^∗∗^*p* < 0.01 relative RNA expression between mature adipocytes treated with vehicle or IL-4, and ^#^*p* < 0.05 between mature adipocytes treated with IL-4 or vehicle.

To determine whether treatment with IL-4 in mature adipocytes could induce browning, precursor cells and mature adipocytes were stimulated with 10 ng/mL IL-4 for 6 h. UCP-1 mRNA was shown to be induced in the presence of IL-4 (Figure 3(a)), and, as shown in Figures 3(b) and 3(c), protein expression levels of PGC-1*α* and CITED1 were increased by 2.3- and nearly 2-fold, respectively.

### 3.4. IL-4 Increases Mitochondrial Protein Activity

**Figure 4:**
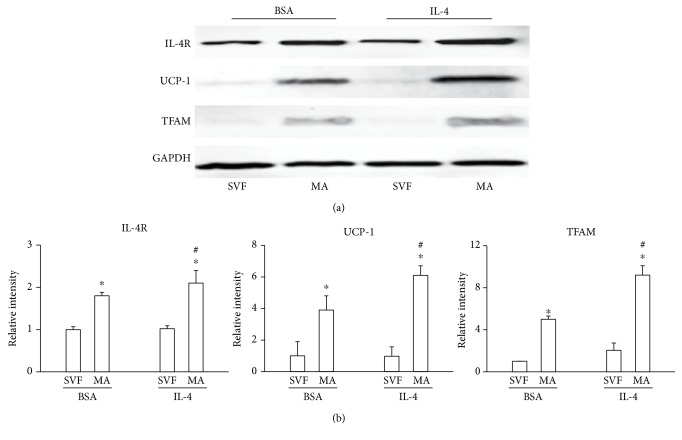
IL-4 treatment increases expression of mitochondrial proteins. Precursors (SVF) were obtained and differentiated as described in Materials and Methods. Cells were treated with BSA alone (vehicle) or IL-4 and incubated at 31°C for 6 h, then lysed. (a) IL-4R, UCP-1, and TFAM protein expression were measured by Western blotting. (b) Relative band intensity of each was determined by densitometry. Statistical analyses were performed using ANOVA. Data represent mean ± SD and were normalized to expression of the reference protein GAPDH. ^∗^*p* < 0.05 between precursors (SVF) and mature adipocytes (MA). ^#^*p* < 0.05 between mature adipocytes (MA) treated with IL-4 or vehicle.

To investigate the effect of IL-4 on mitochondrial activity, mature adipocytes were treated with IL-4 and incubated at 31°C for 6 h. The incubation to 31°C resembles a reduction of temperature and remark the effect on UCP1. Cell lysates were then prepared, and protein expression of UCP1 and TFAM was measured by Western blotting. IL-4 enhanced the hypothermia-induced increases in mitochondrial protein expression (Figures 4(a) and 4(b)), implying a browning effect of IL-4 in mature adipocytes.

### 3.5. Effect of IL-4 on the Expression of Adipokines in Differentiated Adipocytes

**Figure 5:**
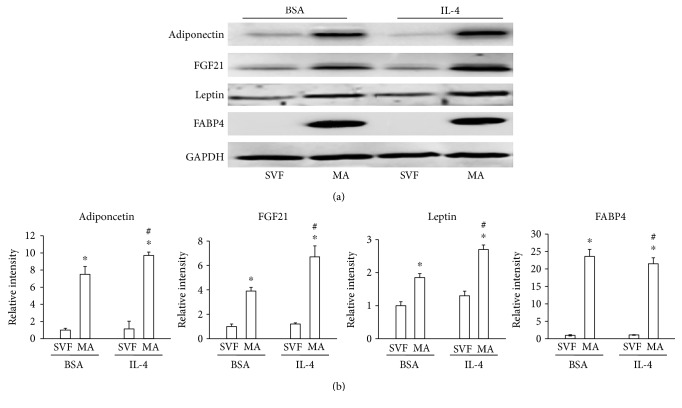
Effect of IL-4 on adipocytokine expression by adipocytes. Precursor cells were obtained, proliferated, and induced to differentiate into mature adipocytes, then treated with BSA alone (vehicle) or IL-4. (a) Adiponectin, FGF21, leptin, and FAB4 protein expression were measured by Western blotting. (b) Relative band intensity of each was determined by densitometry. Statistical analyses were performed using ANOVA. Data represent mean ± SD and were normalized to the reference protein GAPDH. ^∗^*p* < 0.05 between precursors (SVF) and mature adipocytes (MA). ^#^*p* < 0.05 between mature adipocytes (MA) treated with IL-4 and vehicle.

A major challenge in evaluating the induction of browning is the characterization of its effect on the production of adipokines. To assess whether IL-4 treatment of mature adipocytes was capable of inducing proteins involved in lipid and glucose metabolism, the expression of adipokines that influence relevant pathways was assessed. IL-4 increased the expression of adiponectin, leptin, and FGF21 in mature adipocytes compared with cells treated with BSA alone (Figures 5(a) and 5(b)). Expression of FABP4 confirmed that the cells were mature adipocytes.

### 3.6. IL-4 Enhances Hypothermia-Induced Activation of Thermogenesis

**Figure 6:**
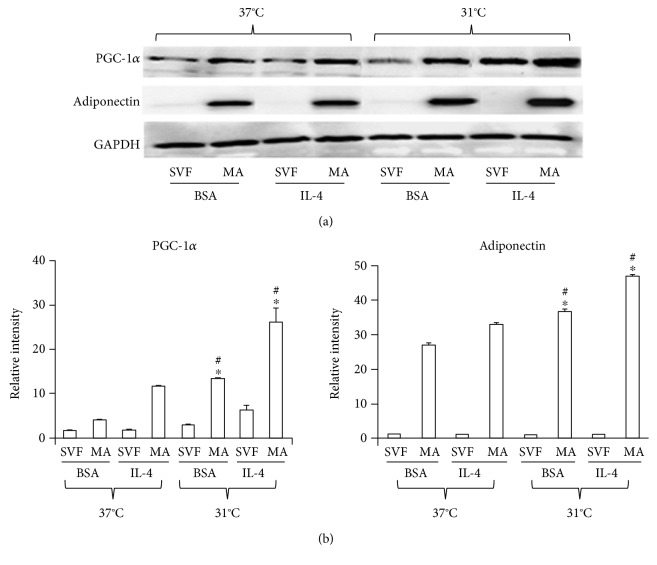
Induction of proteins associated with thermogenesis by cold and IL-4. Precursors were obtained and differentiated as described in Materials and Methods. Cells were treated with BSA alone (vehicle) or IL-4 and incubated at 37°C or 31°C for 6 h. (a) PGC-1*α* and adiponectin protein expression were determined by Western blotting of cell lysates. (b). Relative band intensity of each was determined by densitometry. Statistical analyses were performed using ANOVA. Data represent mean ± SD and were normalized to the reference protein GAPDH. ^∗^*p* < 0.05 between the differentiated adipocytes exposed to 37°C and those exposed to 31°C. ^#^*p* < 0.05 between differentiated adipocytes exposed to 37°C and those exposed to 31°C, in the presence of IL-4.

Adiponectin is an adipokine known for its protective effect against cardiometabolic diseases and diabetes mellitus. Exposure to cold in humans and mice induces the production of adiponectin, in part through activation of the IL-4 pathway. To evaluate whether the IL-4 treatment might increase hypothermia-induced adiponectin expression, adipocytes were exposed to low temperature (31°C) and treated with 10 ng/mL IL-4. This resulted in increased expression of adiponectin and PGC-1*α* in adipocytes, compared to cells exposed to the same conditions of temperature but treated with vehicle (Figures 6(a) and 6(b)).

### 3.7. Lipolytic Effect of IL-4

**Figure 7:**
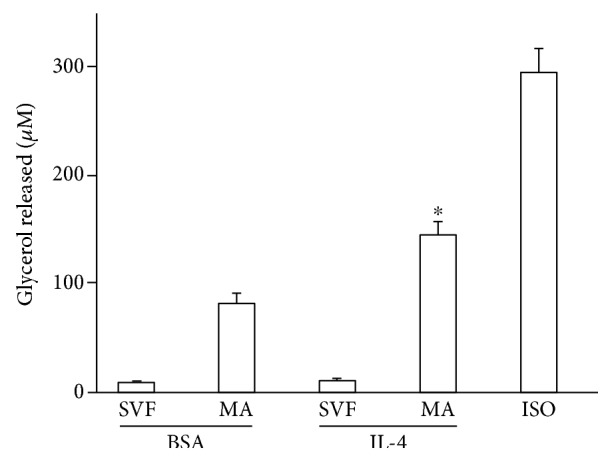
Lipolytic effect of IL-4. Precursor cells were obtained and differentiated as described in Materials and Methods, then treated with BSA alone (vehicle), IL-4, or isoproterenol for 6 or 24 h. The culture media was recovered, and the glycerol concentration was determined. Statistical analyses were performed using ANOVA. Data represent mean ± SD. ^∗^*p* < 0.05 between mature adipocytes treated with vehicle or IL-4.

Our results imply that IL-4 may modulate the phenotype of mature adipocytes by promoting browning and inducing expression of adipokines involved in lipid and carbohydrate metabolism. Therefore, we were prompted to evaluate whether IL-4 could modulate lipid mobilization in the mature adipocyte. To this end, we examined the effect of IL-4 on lipolysis in mature adipocytes and showed that IL-4 treatment for 6 h increased lipolysis (Figure 7).

## 4. Discussion

This work highlights the role of IL-4 receptor (IL-4R) in promoting browning of mature human adipocytes. These adipocyte cells were differentiated from ADMSCs that had been obtained during abdominoplasty in women with normal BMI. IL-4 treatment of the differentiated cells resulted in increased expression levels of pSTAT-6, PGC-1*α*, UCP-1, and CITED1. These findings suggest that IL-4 could be exerting a direct browning effect on adipocytes by binding to IL-4R*α* receptors. Although some studies have considered the role of IL-4 and its receptor in adipocyte browning, these were mainly of adipocyte precursors [11, 23], which preferentially differentiated into beige adipocytes in the presence of IL-4.

Previously, Qiu and colleagues suggested that IL-4 produced by eosinophils stimulated catecholamine production by M2 macrophages, which induced a change in adipocyte phenotype towards that of beige adipocytes [14]. Other studies showed that ILC2s do not require IL-4 to induce browning; instead, they may induce this effect by increased met-enkephalin production after stimulation with IL-33 [10]. Recently, it was observed that during the winter season the browning process increases, an event mediated by IL-4 and mast cells [24]. The present work showed that IL-4R in humans and pSTAT-6 are expressed in differentiated adipocytes and that IL-4 treatment increases expression of PGC-1*α*, UCP-1, and CITED1.

In addition, IL-4 treatment increases expression levels of adipokines in adipocytes, including those of adiponectin and FGF21 [25, 26], in which the effects are metabolically beneficial. This finding is relevant because, in addition to increasing thermogenesis in adult adipocytes, IL-4 may have an additional favorable effect on carbohydrate metabolism. Both adiponectin and FGF21 may improve glucose uptake by peripheral tissues and insulin sensitivity [27, 28].

**Figure 8:**
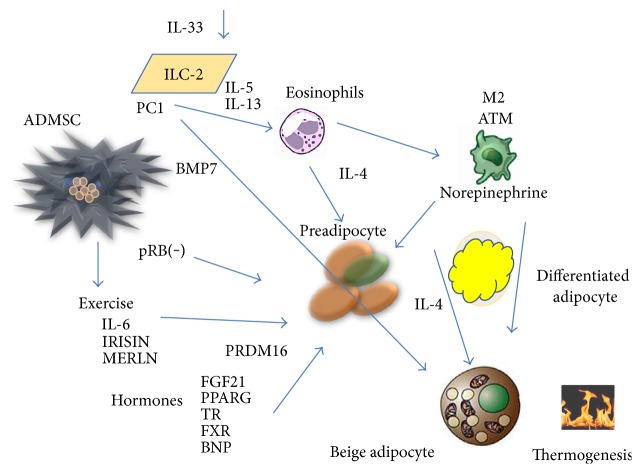
Determining factors in differentiation of beige adipose cell in humans. The adipose mesenchymal cells can be influenced by the retinoblastoma protein (pRb) and take the decision to differentiate into fat cells when pRb is blocked. BMP7 triggers production of mesenchymal adipose cells to brown adipose cells. Both exercise and some hormones can increase the capacity of adipose stem cells to differentiate into beige adipocytes. Recently, it has been observed that cells of the innate immune system type 2 can secrete interleukins stimulating the production of IL-4 by eosinophils and norepinephrine production through macrophage type 2. IL-33 can activate the differentiation of beige adipocytes directly. ADMSC: adipose mesenchymal stem cell; PC1: Prohormone Convertase 1; ILC-2: group 2 innate lymphoid cells; BMP7: bone morphogenic protein 7; M2 ATM: adipose tissue macrophage 2; FGF21: fibroblastic growth factor 21; PPAR*γ*: peroxisome proliferator-activated receptor gamma; TR: thyroid receptors; FXR: farnesoid X receptor; BNP: brain natriuretic factor.

The effect of IL-4 on the expression of proteins implicated in thermogenesis in mature adipocytes was accompanied by an increase in lipolysis shown by increased glycerol release into the medium (Figure 5). A further interesting observation was the increased expression levels of UCP-1, TFAM, PGC-1*α*, and adiponectin when the cells were subjected to low temperature, as shown in Figures 4 and 6. The response of the cells to cold stress and the mechanism for acclimatization to environmental cold are mediated in part by type 2 immune cells (ILC2s, eosinophils, and activated macrophages) that produce interleukins (IL-4, IL-13, and IL-33) [11]. It was previously demonstrated that cold can enable the induction of a beige phenotype in cultured adipocytes, which is mediated by IL-4, among other factors [16, 29]. Our observations in the present work showed that further increase in the thermogenic proteins observed with temperature reduction exhibits additional mechanisms than the only effect of IL-4 and IL-4R. Other factors that may contribute to the outcome of temperature reduction on the increase in thermogenic proteins in the adipocytes can be the activation of adrenergic receptors and methionine-enkephalin [30–32]. However, more research is required to address this question. Given that previous studies that observed reductions in IL-4R expression were performed in mouse and rat adipocyte cell lines, we believe that this study is novel in demonstrating the involvement of IL-4 and its receptor in the browning of mature adipocytes derived from human ADMSCs. It will be important to analyze the metabolic behavior of patients who are treated with specific blockers of IL-4R in therapies for atopic diseases. These data imply that targeting IL-4R to encourage increased energy expenditure by adipocytes may be of use in the treatment of obesity. A schematic role of ILs in the browning process is shown in Figure 8.
